# Eradication of *Potato Virus S*, *Potato Virus A*, and *Potato Virus M* From Infected *in vitro*-Grown Potato Shoots Using *in vitro* Therapies

**DOI:** 10.3389/fpls.2022.878733

**Published:** 2022-05-19

**Authors:** Jean Carlos Bettoni, Liya Mathew, Ranjith Pathirana, Claudia Wiedow, Donald A. Hunter, Andrew McLachlan, Subuhi Khan, Joe Tang, Jayanthi Nadarajan

**Affiliations:** ^1^The New Zealand Institute for Plant and Food Research Limited, Food Industry Science Centre, Palmerston North, New Zealand; ^2^Plant Health and Environment Laboratory, Ministry for Primary Industries, Auckland, New Zealand

**Keywords:** cryotherapy, thermotherapy, chemotherapy, high-health plants, shoot tips, cryopreservation, microtubers, *Solanum tuberosum*

## Abstract

Certain viruses dramatically affect yield and quality of potatoes and have proved difficult to eradicate with current approaches. Here, we describe a reliable and efficient virus eradication method that is high throughput and more efficacious at producing virus-free potato plants than current reported methods. Thermotherapy, chemotherapy, and cryotherapy treatments were tested alone and in combination for ability to eradicate single and mixed *Potato virus S* (PVS), *Potato virus A* (PVA), and *Potato virus M* (PVM) infections from three potato cultivars. Chemotherapy treatments were undertaken on *in vitro* shoot segments for four weeks in culture medium supplemented with 100 mg L^−1^ ribavirin. Thermotherapy on *in vitro* shoot segments was applied for two weeks at 40°C (day) and 28°C (night) with a 16 h photoperiod. Plant vitrification solution 2 (PVS2) and cryotherapy treatments included a shoot tip preculture followed by exposure to PVS2 either without or with liquid nitrogen (LN, cryotherapy) treatment. The virus status of control and recovered plants following therapies was assessed in post-regeneration culture after 3 months and then retested in plants after they had been growing in a greenhouse for a further 3 months. Microtuber production was investigated using *in vitro* virus-free and virus-infected segments. We found that thermotherapy and cryotherapy (60 min PVS2 + LN) used alone were not effective in virus eradication, while chemotherapy was better but with variable efficacy (20–100%). The most effective result (70–100% virus eradication) was obtained by combining chemotherapy with cryotherapy, or by consecutive chemotherapy, combined chemotherapy and thermotherapy, then cryotherapy treatments irrespective of cultivar. Regrowth following the two best virus eradication treatments was similar ranging from 8.6 to 29% across the three cultivars. The importance of virus removal on yield was reflected in “Dunluce” free of PVS having higher numbers of microtubers and in “V500’ free of PVS and PVA having a greater proportion of microtubers > 5 mm. Our improved procedure has potential for producing virus-free planting material for the potato industry. It could also underpin the global exchange of virus-free germplasm for conservation and breeding programs.

## Introduction

Potato (*Solanum tuberosum*) is the world’s third most important food crop after rice and wheat, and is the staple food of 1.3 billion people (Devaux et al., 2021). In 2019, the planted area of potatoes, globally, reached 17.3 million hectares, producing of over 370 million tons of which 45% was produced in China (25%), India (14%), and Russia (6%; FAOSTAT-Food and Agriculture Organization of the United Nations, 2021). In New Zealand, potato is a valuable commodity crop with 497,634 MT produced from 9,775 ha: in 2020, this production was valued at NZ$ 1.1 billion, including a significant export value close to NZ$ 106 million (Potatoes NZ, 2021). At the New Zealand Institute for Plant and Food Research Ltd. (PFR), we maintain field collections covering both old and new cultivars that have been used in breeding programs. A subset of these are held in tissue culture and a cryopreservation program to secure the collection is underway (Pathirana et al., 2019), with over 300 lines already secured.

High-quality seed potato is crucial for successful potato cultivation (Onofre et al., 2021). Viral diseases are one of the primary reason for degeneration of seed potatoes and thus constitute a major constraint for sustainable potato production (Awasthi and Verma, 2017; Priegnitz et al., 2019; Wasilewska-Nascimento et al., 2020). Vegetative propagation of potato results in virus transmission from generation to generation, with virus titers accumulating as a result of repeated propagation and infection events (Thomas-Sharma et al., 2016; Priegnitz et al., 2020). As well as inducing increased susceptibility to other pathogens, viral diseases cause economic losses due to their negative impacts on yield and tuber quality (Lin et al., 2014; Adolf et al., 2020).

Although around 57 viruses have been identified as infecting cultivated potatoes, only a few are reported to have a major economic impact. *Potato virus M* (PVM; *Carlavirus*), *Potato virus A* (PVA; *Potyvirus*), and *Potato virus S* (PVS; *Carlavirus*) are typically the most significant viral pathogens (either as single or as mixed infections) associated with substantial production losses (Wang et al., 2011; Fletcher, 2012; Kreuze et al., 2020). These virus species are disseminated through tubers and by aphids in a non-persistent manner. PVS and PVA are also transmitted by contact (Valkonen, 2007; Fuentes et al., 2021). Plants infected by PVM and PVS may not always show symptoms depending on the cultivar and virus isolates (Kreuze et al., 2020; Bradshaw, 2021). Furthermore, mixed infection can increase symptom severity and virus accumulation (Nyalugwe et al., 2012; Moreno and López-Moya, 2020).

Production of virus-free plants is necessary for successful management of viral diseases and for sustainable breeding activities including preservation of potato germplasm and global exchange of genetic resources (Naik and Khurana, 2003; Volmer et al., 2017; Ellis et al., 2020). Besides complete eradication, there are no effective measures for controlling viruses once the plants are infected (Nazarov et al., 2020; Rubio et al., 2020). Therefore, there is a need to develop efficient methods to eradicate virus species to ensure production and supply of high-healthy planting material for the potato industry. Various *in vitro*-based techniques have been used to eradicate viruses in potato plants. For example, meristem culture used either alone (Quazi and Martin, 1978; Wang et al., 2006; Zhang et al., 2019) or in combination with thermotherapy (Wang et al., 2006; AlMaarri et al., 2012; Waswa et al., 2017), chemotherapy (AlMaarri et al., 2012), and electrotherapy (AlMaarri et al., 2012). In meristem culture-based methods, the size of the explant affects the efficacy of virus eradication; it is usually necessary to excise shoot tips of 0.2 mm containing an apical dome with one or two leaf primordia (Wang et al., 2006; Zhang et al., 2019). Excision of such small shoot tips is laborious, time-consuming, and a highly skilled task: results can also be variable in terms of shoot regrowth and the frequency of virus eradication (Bettoni et al., 2016; Magyar-Tábori et al., 2021). The inability to guarantee complete removal of viral particles, especially in mixed infections, remains a limitation for these meristem culture-based methods (Faccioli and Marani, 1998; Zhang et al., 2019). Application of shoot tip cryotherapy as a novel method for plant virus eradication has also been tested in potato with mixed results (Wang et al., 2006; Jianming et al., 2012; Yi et al., 2014; Li et al., 2016, 2018a; Zhang et al., 2019). For example, *Potato leafroll virus* (PLRV) and *Potato virus Y* (PVY) have been successfully eliminated with high efficacy using cryogenic treatments (Wang et al., 2006; Yi et al., 2014; Zhang et al., 2019); however, this method completely failed in producing PVM- and PVS-free potato plants when they presented as a mixed infection (Li et al., 2018a; Zhang et al., 2019).

Improved potato virus eradication has been achieved by combining two or more *in vitro*-based techniques. Combining chemotherapy with thermotherapy (Fletcher et al., 1988; Fang et al., 2005; Dhital et al., 2007; Nasir et al., 2010; Antonova et al., 2017) and meristem culture (Zhang et al., 2019) or chemotherapy with cryotherapy (Kushnarenko et al., 2017) were shown to provide more effective virus eradication than just a single technique. However, the efficacy of these methods varied according to the virus species, their infection level (single or mixed, virus titer) and cultivars used, as well as the virus-host combination (Antonova et al., 2017; Waswa et al., 2017; Gong et al., 2019). Furthermore, duration and temperature of the thermotherapy treatment, types, and concentrations of antiviral agents used in chemotherapy, as well as the size of the excised shoot tip can affect the success of virus eradication (AlMaarri et al., 2012; Kushnarenko et al., 2017; Waswa et al., 2017; Magyar-Tábori et al., 2021). Therefore, standardization of the virus eradication methodology is important, especially when the plants have mixed infections.

Herein, we investigated the effect of chemotherapy, thermotherapy, and cryotherapy on eradication of PVM, PVA, and PVS from *in vitro* cultured shoots of three potato cultivars. More specifically, we combined several of these methods to develop a novel technology that is high throughput, easier, and more effective in eradicating virus. We believe that this technology can be easily adopted and transferred between laboratories, facilitating delivery of healthy propagating material to growers for commercial production and for germplasm conservation and plant exchange. Data demonstrating the positive effect that removal of these viruses has on microtuber yield are also presented.

## Materials and Methods

### Plant Material and Explant Preparation for *in vitro* Therapies

Tissue-cultured plants of three potato (*Solanum tuberosum* L.) cultivars “Dunluce,” “Tahi,” and “V500” were obtained from the Lincoln site of PFR and used in this study. Infected *in vitro* stock plants with PVM, PVA, and PVS were previously identified and confirmed using the reverse-transcription polymerase chain reaction (RT-qPCR) method (see below) prior to the experiments outlined here. Virus-infected cultures (“Dunluce” with PVS, “Tahi” with PVA and PVS, and “V500” with PVM and PVS) were multiplied and maintained in an actively growing state on basal medium (BM) composed of half-strength Murashige and Skoog (1962; MS) macronutrients, full-strength MS micronutrients, and Linsmaier and Skoog (1965; LS) vitamins supplemented with 30 g L^−1^ sucrose and 7.5 g L^−1^ agar. For all experiments, the pH of culture media was adjusted to 6 prior to autoclaving at 121°C for 20 min. Cultures were incubated in a growth room maintained at 24°C with a 16 h photoperiod at a photosynthetic flux density of 40 μmol s^−1^ m^−2^ provided by cool-white fluorescent tubes (hereafter called standard conditions). Subculture into fresh BM was performed every 4 weeks throughout the experiment.

**Figure 1:**
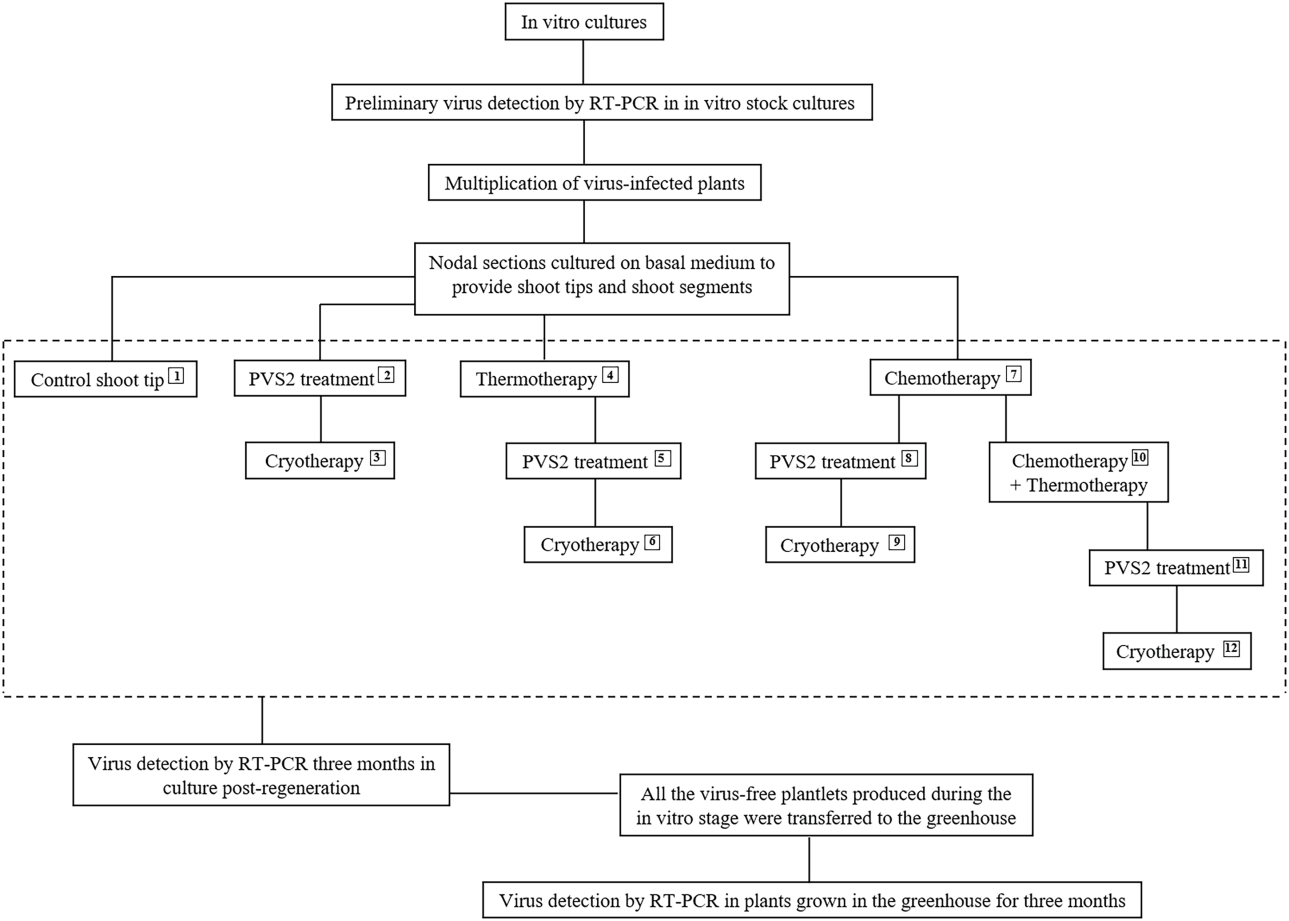
Flowchart depicting the 12 *in vitro* therapies used for eradicating *Potato virus S*, *Potato virus A*, and *Potato virus M* from infected *in vitro*-grown potato shoots. Plant vitrification solution 2 (PVS2) and cryotherapy treatments were performed on 1 mm apical shoot tips and thermotherapy and chemotherapy on 1–1.5 cm apical shoot segments.

The explants used for *in vitro* therapies were nodal sections (1 cm in length) obtained from 3-week-old *in vitro* stock plants and placed on 90 × 15 mm disposable polystyrene Petri plates containing 25 ml of BM at a density of 30 to 40 nodal sections per plate. The nodal sections were cultured under standard conditions to provide uniform explant material for the therapies. The apical shoot tips (1 mm) for cryotherapy were excised from the nodal sections after 1 week, and the shoots (1.5 cm) for chemotherapy/thermotherapy experiments were excised from the nodal sections after 2 weeks. Figure 1 shows the workflow for this study.

### *In vitro* Therapies for Virus Eradication

#### Control Shoot Tips (SP)

Uniform apical shoot tips (1 mm length) were excised from 1-week-old shoots and cultured on recovery medium (RM) consisting of MS macro and micro elements, LS vitamins, 25 g L^−1^ sucrose, 0.05 mg L^−1^ indole-3-acetic acid, 0.05 mg L^−1^ gibberellic acid, 0.3 mg L^−1^ zeatin, and 6 g L^−1^ agar at pH 5.7; (Kim et al., 2006 with modifications) and incubated in the dark at 24°C for 1 week before transfer to standard conditions.

#### Plant Vitrification Solution 2 Treatment (PVS2)

Uniform apical shoot tips (1 mm length) were excised from 1-week-old shoots and transferred to liquid preculture medium 1 (half-strength MS macronutrients, MS micronutrients, and LS vitamins containing 0.3 M sucrose) and maintained for 24 h on a shaker (50 rpm) in darkness at 24°C. They were then transferred to liquid preculture medium 2 (same salts as preculture medium 1 but with 0.7 M sucrose) and maintained under the same conditions for another 16 h. Precultured shoot tips were treated with plant vitrification solution 2 (PVS2) [filter-sterilized MS, 0.4 M sucrose, 30% (w/v) glycerol, 15% (w/v) ethylene glycol (EG), and 15% (w/v) dimethyl sulfoxide (DMSO), pH 5.8] (Sakai et al., 1990) at 22°C for 60 min (PVS2 treatment).

Following PVS2 treatment, the shoot tips were washed with unloading solution (filter-sterilized half-strength MS macronutrients, MS micronutrients + 1.2 M sucrose at pH 5.8) pre-warmed to 40°C and maintained in the solution for 20 min at room temperature (22°C). Thereafter, for recovery, shoot tips were transferred to an intermediate recovery medium (half-strength MS macronutrients, MS micronutrients supplemented with 0.6 M sucrose, and 7.5 g L^−1^ agar) overnight in the dark and then transferred to RM. Cultures were maintained 1 week in the dark at 24°C, before being transferred to standard conditions.

Additionally, the survival, shoot regrowth, and virus eradication efficiency from shoot tips after treatment with one of seven PVS2 exposure periods between 5 and 135 min in “Dunluce” and after short, medium, and long PVS2 exposure periods (5, 60, and 135 min, respectively) in “Tahi” and “V500” were assessed.

#### Cryotherapy

For cryotherapy (Cryo), similar steps to those described in “PVS2” were followed, and 2 minutes before the end of PVS2 incubations, PVS2-treated shoot tips were placed into a thin layer of PVS2 on sterile aluminum foil strips (~ 6 × 25 mm) and then plunged into LN. After 1 hour of LN exposure, the aluminum foil strips with shoot tips were thawed quickly by inverting the strips into unloading solution and recovered as described in “PVS2” above.

#### Thermotherapy (T)

Apical shoot segments (1–1.5 cm in length) were excised from 2-week-old shoots and placed in 98 × 60 mm disposable polystyrene clear tissue culture vessels with 50 ml BM at a density of 15 nodal sections per culture vessel. After 2 days of culture under standard conditions, the culture vessels containing shoot segments were moved into a growth chamber with 40% relative humidity at a photosynthetic photon flux density of 70 μmol s^−1^ m^−2^ provided by cool-white fluorescent tubes. The shoots were grown in an alternating temperature regime of 28°C for 8 h in darkness and 40°C for 16 h with light for 2 weeks. Apical shoot tips (1 mm length) were excised from heat-treated shoots and cultured on RM in the dark at 24°C for 1 week before transfer to standard conditions.

#### Thermotherapy + PVS2 (T + PVS2)

Apical shoot segments (1–1.5 cm in length) were subjected to thermotherapy, as described in “Thermotherapy”, after which apical shoot tips (1 mm length) were excised and subjected to PVS2 treatment (PVS2) as described in “PVS2”.

#### Thermotherapy + Cryotherapy (T + Cryo)

Apical shoot segments (1–1.5 cm in length) were subjected to thermotherapy, as described in “Thermotherapy”, after which apical shoot tips (1 mm length) were excised and subjected to cryotherapy (Cryo) as described in “Cryotherapy”.

#### Chemotherapy (C)

Apical shoot segments (1–1.5 cm in length) were excised from 2-week-old shoots and placed in 98 × 60 mm disposable polystyrene clear tissue culture vessels containing 50 ml BM supplemented with 100 mg L^−1^ ribavirin (Duchefa^®^, Haarlen, Netherlands) at a density of 15 nodal sections per culture vessel. Ribavirin was filter-sterilized and added to the medium after autoclaving. The cultures were maintained under standard conditions. After 4 weeks of ribavirin treatment, apical shoot tips (1 mm length) were excised and placed on RM and incubated in the dark at 24°C for 1 week before transfer to standard conditions.

#### Chemotherapy + PVS2 (C + PVS2)

Apical shoot segments (1–1.5 cm in length) were cultured on BM supplemented with 100 mg L^−1^ ribavirin for 4 weeks under standard conditions, as described in “Chemotherapy”, after which apical shoot tips (1 mm length) were excised and subjected to PVS2 treatment (PVS2) as described in “PVS2”.

#### Chemotherapy + Cryotherapy (C + Cryo)

Apical shoot segments (1–1.5 cm in length) were cultured on BM supplemented with 100 mg L^−1^ ribavirin for 4 weeks under standard conditions, as described in “Chemotherapy”, after which apical shoot tips (1 mm length) were excised and subjected to cryotherapy (Cryo) as described in “Cryotherapy”.

#### Chemotherapy Followed by Combined Chemotherapy and Thermotherapy [C + (C + T)]

Apical shoot segments (1–1.5 cm in length) were cultured on BM supplemented with 100 mg L^−1^ ribavirin for 2 weeks under standard conditions, as described in “Chemotherapy”, after which the tissue culture vessel containing the shoots in ribavirin medium was transferred to thermotherapy conditions as described in “Thermotherapy” for additional 2 weeks, followed by apical shoot tip isolation (1 mm length) and recovery process as described in “Thermotherapy”.

#### Chemotherapy Followed by Combined Chemotherapy and Thermotherapy, Then PVS2 [C + (C + T) + PVS2]

Apical shoot segments (1–1.5 cm in length) were treated as described in “Chemotherapy Followed by Combined Chemotherapy and Thermotherapy [C + (C + T)]” above, after which apical shoot tips (1 mm length) were excised and subjected to PVS2 treatment (PVS2), as described in “PVS2”.

#### Chemotherapy Followed by Combined Chemotherapy and Thermotherapy, Then Cryotherapy [C + (C + T) + Cryo]

Apical shoot segments (1–1.5 cm in length) were treated as described in “Chemotherapy Followed by Combined Chemotherapy and Thermotherapy [C + (C + T)]”, after which apical shoot tips (1 mm length) were excised and subjected to cryotherapy (Cryo), as described in “Cryotherapy”.

### Shoot Tip Recovery

**Figure 2:**
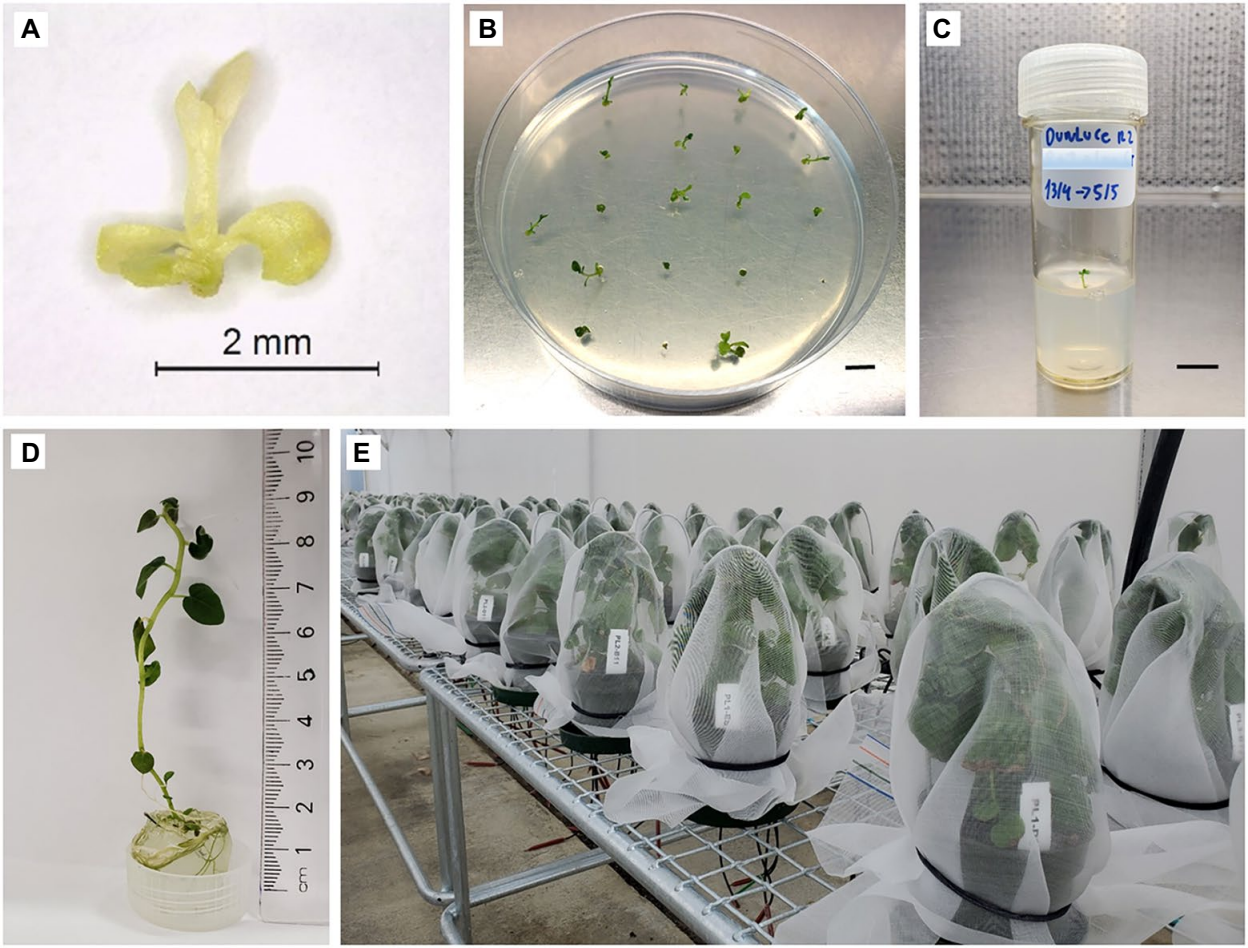
Shoot tip recovery process in potato “Dunluce” following a combined chemotherapy + cryotherapy treatment. **(A)** Shoot tip 1 week after combined chemotherapy + cryotherapy and **(B)** 3 weeks recovery from cryoexposure. **(C)** Shoot transferred to vial and **(D)** grown for 3 months. **(E)** Plants after 3 months of growth in the greenhouse. Bars = B 0.6 cm, C 0.7 cm.

Shoot tip survival (shoot tips that exhibited growth of a green cell mass or leaf tissue; Figure 2A) and regrowth (shoot tips exhibiting organized shoots with a new leaf emerging; Figure 2B) were recorded 3–6 weeks post-culture on RM. Regenerated shoots with approx. 1 cm of growth were transferred to individual vials (30 ml capacity) with 10 ml of BM and grown under standard conditions (Figures 2B,C).

### Virus Detection

The virus status of plant materials was tested using RT-qPCR. Primers and probes (Table 1) were purchased from Integrated DNA Technologie.^1^ The potato mitochondrial NADH dehydrogenase subunit 5 (*nad5*) gene transcript (Khan et al., 2015) and the plant nuclear gene transcript exocyst complex component (*sec3*) were used as the plant positive controls for cDNA synthesis. RT-qPCR was performed on nucleic acids isolated from young leaves of tissue-cultured plantlets and greenhouse-grown plants by the commercial company SlipStream Automation^2^ using their standard in-house CTAB- and plate-based extraction and cDNA synthesis methodology. cDNA generated using SuperScriptIV^3^ from the RNA was diluted 5-fold with TE buffer (pH 8.0) for use in PCR. PCRs for PVS, PVA, PVM, and *nad5* were performed in 5.5 μl volumes containing 0.9 x Roche Lightcycler 480 Master Mix,^4^ 680 nM primers, 227 nM probe, and 2 μl diluted cDNA. Prior to amplification, cDNA was denatured at 95°C for 4 min and then cycled 40 times with 95°C/15 s and 60°C/45 s. Samples were scored with the LightCycler 480 software release 1.5.1.62 SP2 using the “Abs Quant/Fit Points” analysis with the Noise Band fixed at a fluorescence value of 3, 1.5, and 3 for PVA, PVM, and PVS, respectively. Samples with viral Ct values greater than 35 were interpreted as absent for virus. PCRs for *sec3* were performed in 7 μl reactions using 1 x Roche Lightcycler 480 Master Mix, 3.12 mM Mg^2+^, 400 nM primers, and 2 μl cDNA. Prior to amplification, the cDNA was denatured at 95°C for 10 min and then cycled 45 times with 95°C/15 s, 60°C/15 s, and 72°C/30s. Following PCR, melt curve analysis was performed at 95°C/60s and 40°C/60s, followed by heating to 65°C and then to 95°C with continuous data acquisition at 25 acquisitions per degree Celsius. Results were scored with LightCycler 480 software release 1.5.1.62 SP2 using the “Abs Quant/Fit Points” analysis with the Noise Band fixed at a fluorescence value of 5.

**Table 1:** Primers and probes used for detection of PVS, PVA, PVM, plant mitochondrial gene transcript NADH dehydrogenase subunit 5 (*nad5*), and plant nuclear gene transcript exocyst complex component (*sec3*).

Primer/probe	Sequence	Amplicon size (bp)	References
NAD5-TM-F	GCTTCTTGGGGCTTCTTGTT		Khan et al. (2015) (modified from Menzel et al., 2002)
NAD5-TM-R	CCAGTCACCAACATTGGCATAA	176	Khan et al. (2015) (modified from Menzel et al., 2002)
NAD5-P	/56-FAM/AGGATCCGC/ZEN/ATAGCCCTCGATTTATGTG/3IABkFQ/		Botermans et al. (2013)
sec3_F	GCTTGCACACGCCATATCAAT		Tang et al. (2017)
sec3_R	TGGATTTTACCACCTTCCGCA		Tang et al. (2017)
PVS-F	TTGACACATTCGATTATGTGAC		This study
PVS-R	GTGATTGCGCACAATCTCAGC		
PVS-P1^*^	FAM-ATGGCAATTGACAAGTCGAACAGAAATG-BHQ-1		
PVS-P2^*^	FAM-AGGAGACGATAGCTCATAACGCTCACAA-BHQ-1		
PVA-F	TGTCGATTTAGGTACTGCTGGGAC		Agindotan et al. (2007)
PVA-R	TGCTTTGGTTTGTAAGATAGCAAGTG		
PVA-P	FAM-CACTACCAATGCTCAAAGGTAAGAGTGTCG-BHQ-1		
PVM-F	AGGTGTCACAGGTGCTATCGC		This study
PVM-R	TCACCTCGGTTACTCCTTCATC		
PVM-P	6FAM-CGCCACGCGCACATTGTA-MGBNFQ		

* PVS-P1 and PVS-P2 probes are used to detect all PVS strains.

PVM, Potato virus M, PVA, Potato virus A, PVS, Potato virus S, and bp, base pairs.

Plantlet material was confirmed to have single or mixed virus infections prior to therapies by RT-qPCR. RT-qPCR-based diagnostic of stock plants revealed the presence of single virus infection of PVS for the cultivar “Dunluce,” PVS and PVA for “Tahi,” and PVS and PVM for “V500.” The presence of viruses in potato cultivars that underwent the *in vitro* therapies and controls were assessed at two different stages: firstly of cultures post-regeneration, 3 months after therapy treatments (Figure 2D), and secondly on all samples with initial negative test results after growing plants on the greenhouse for a further 3 months (Figure 2E).

### *In vitro* Rooting, Acclimatization, and Plant Maintenance in the Greenhouse

One apical shoot segment (1.5 cm in length), containing two to three fully expanded *in vitro* leaves, from each virus-free plant was cultured in an individual vial (30 ml capacity) on BM under standard conditions for 2 weeks to initiate rooting. The rooted plantlets were then transferred to individual 12-cm diameter pots (800 ml capacity) containing a commercial substrate Daltons® potting mix (Daltons, Matamata, New Zealand) in an insect-proof greenhouse at 22 ± 2°C with a 12–14 h photoperiod under natural light. Plants were individually covered with insect-proof net to prevent possible virus-cross contamination (Figure 2E). After 3 months of growth (September to November), leaves were collected for further confirmation of the sanitary status by RT-qPCR.

### Assessment of the Impact of Virus Infection on Microtuber Production

**Figure 3:**
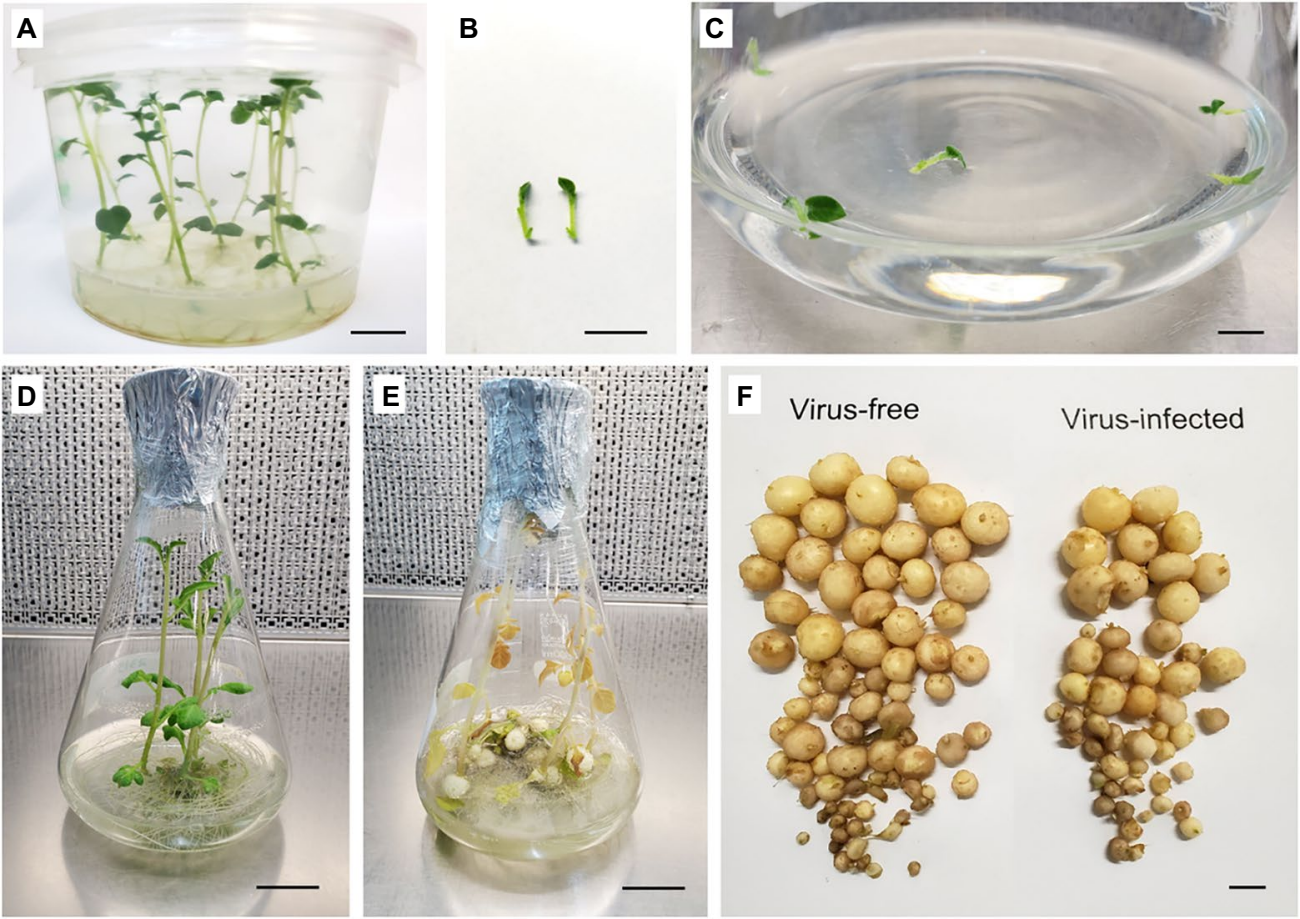
Microtuber production in *in vitro* cultures of potato cultivar “Dunluce.” **(A)** 3-week-old *in vitro* stock plants. **(B)** Shoot segments (1 cm) used as the explant source for microtuber production. **(C)** Shoot segments after initiation and **(D)** growth after 14 days of liquid culture at 24°C with a photoperiod of 16 h light day^–1^. **(E)** Cultures after 14 days in microtuber production medium at 24°C in the dark. **(F)** Microtubers produced on shoots derived from virus-free (left) and *Potato Virus S*-infected (right) *in vitro* plants of “Dunluce” potato. Bars = A, B, C, and F 1 cm; D and E 2.3 cm.

*In vitro* microtubers were induced using the method of Wakasa et al. (2020) with modifications as follows. Apical shoot segments (1 cm length) containing a single leaf and one to two axillary buds were obtained from 3-week-old *in vitro* virus-free and virus-infected stock plants. Five shoot segments were placed into 100 ml liquid BM in a 500-mL Erlenmeyer flask and were cultured on a shaker (75 rpm) under standard conditions for 2 weeks (Figures 3A–D). The BM was then discarded and replaced with liquid microtuber production medium (MPM; Li et al., 2013; Zhang et al., 2019) and grown in the dark at 24°C. The MPM is composed of BM supplemented with 60 g L^−1^ sucrose and 4 mg L^−1^ kinetin. Data on the number of microtubers produced per vessel and the size of microtubers (diameter) were recorded after 2 weeks of culture in liquid MPM (Figures 3E,F).

### Data Analysis

A minimum of 20 shoot tips were used per replicate with three replicates per treatment in a randomized block design. Shoot tip survival and regrowth results were both analyzed using binomial generalized linear models (GLM) with logit link (logistic regression). For each treatment, the results were presented as the number of samples, the percentage success, and a 95% confidence interval for the percentage. Confidence limits for the predicted treatment means were calculated from the logistic regression results except for those means of 100% for which the confidence limits were replaced by limits calculated using the Wilson Score method (Agresti and Coull, 1998; Supplementary Tables 1–5). Comparisons between treatment means were made using pairwise contrasts.

The numbers of microtubers produced per vessel from virus-free and virus-infected sources were compared using one-way ANOVA separately for each cultivar., and the proportions of microtubers in each size class (<5 mm, 5–10 mm, >10 mm) were analyzed using a binomial GLM with a logit link separately for each cultivar and size class (Supplementary Tables 6, 7). All analyses were performed using Genstat 19th edition (VSN International, Hemel Hempstead, United Kingdom). Frequency of virus eradication was calculated as the percentage of virus-free plants over the total number of plants used for virus detection. Up to 10 plantlets recovered from *in vitro* therapies and controls were randomly selected from the population and virus tested using RT-qPCR. The total number of plantlets used for virus testing varied depending on the number of samples obtained in each treatment.

## Results

### Effects of the *in vitro* Therapies on Shoot Tip Viability and Virus Eradication

All the shoot tips in the controls survived and at least 96.8% regenerated shoots (Table 2). All the regenerated plants from the virus-infected positive controls remained virus-infected (Tables 3). After 2 weeks of thermotherapy alone or thermotherapy in combinations with chemotherapy, shoots developed a yellowish coloring at the tip. Despite this possible sign of stress, there was no significant impact on shoot tip regrowth of cultivar “Dunluce”; however, shoot tip regrowth of cultivars “Tahi” and ‘V500” was reduced compared to the control (Table 2). For “V500,” all the thermo- and chemo-treated shoot tips survived, but only a proportion produced viable plants (50.8 and 48.3%, respectively) with most shoot tips having yellowish growth that failed to develop (Table 2; Supplementary Tables 1, 2).

**Table 2:** Survival and regrowth levels (%) of shoot tips excised from *in vitro*-grown potato cultivars “Dunluce,” “Tahi,” and “V500” following each treatment of virus eradication.

**Treatment**	“**Dunluce**”			“**Tahi**”			“**V500**”		
	***N***	**Survival (%)**	**Regrowth (%)**	***N***	**Survival (%)**	**Regrowth (%)**	***N***	**Survival (%)**	**Regrowth (%)**
ST (control)	62	100 a	96.8 a	61	100 a	98.4 a	60	100 a	100 a
PVS2	61	98.4 ab	73.8 b	63	100 a	58.7 d	65	96.9 ab	58.5 b
Cryo	74	94.6 bc	55.4 c	70	77.1 c	44.3 de	64	93.8 bc	42.2 bcde
T	62	100 a	93.5 a	60	98.3 ab	80 bc	61	100 a	50.8 bc
T + PVS2	72	95.8 abc	47.2 c	72	94.4 b	43.1 de	63	87.3 cd	33.3 def
T + Cryo	78	67.9 ef	12.8 e	63	76.2 c	11.1 g	76	80.3 d	23.7 f
C	60	98.3 ab	90 a	60	100 a	80 bc	60	100 a	48.3 bcd
C + PVS2	67	88.1 cd	56.7 c	67	92.5 b	40.3 e	70	95.7 abc	41.4 cde
C + Cryo	80	55 f	28.8 d	70	58.6 d	8.6 g	76	80.3 d	19.7 f
C + (C + T)	61	98.4 ab	88.5 a	60	100 a	81.7 b	60	100 a	41.7 bcde
[C + (C + T)] + PVS2	66	78.8 de	48.5 c	64	93.8 b	65.6 cd	60	100 a	45 bcde
[C + (C + T)] + Cryo	75	58.7 f	10.7 e	74	56.8 d	17.6 fg	69	63.8 e	29 f

*N*, number of shoot tips used for each treatment; ST, shoot tip; Cryo, cryotherapy (liquid nitrogen treatment); PVS2, plant vitrification solution 2 (without liquid nitrogen exposure); T, thermotherapy; and C, chemotherapy. Different letters in the same column indicate significant differences at *p* < 0.05 according to pairwise contrasts.

**Table 3:** Effect of virus eradication treatments on percentage of potato plants from cultivars “Dunluce,” “Tahi,” and “V500” that were virus-free in a single or mix-infection.

**Treatment**	“**Dunluce**”	“**Tahi**”	“**V500**”		
	**Virus-free plants (%)**	**Virus-free plants (%)**			**Virus-free plants (%)**		
	**PVS**	**PVS**	**PVA**	**PVS- and PVA-free**	**PVS**	**PVM**	**PVS- and PVM-free**
ST (control)	0 (0/10)	0 (0/10)	0 (0/10)	0 (0/10)	0 (0/10)	0 (0/10)	0 (0/10)
PVS2	0 (0/10)	0 (0/10)	0 (0/10)	0 (0/10)	0 (0/10)	0 (0/10)	0 (0/10)
Cryo	0 (0/10)	0 (0/10)	20 (2/10)	0 (0/10)	0 (0/10)	0 (0/10)	0 (0/10)
T	0 (0/10)	0 (0/10)	70 (7/10)	0 (0/10)	30 (3/10)	20 (2/10)^*^	20 (2/10)^*^
T + PVS2	20 (2/10)	20 (2/10)	70 (7/10)	20 (2/10)	20 (2/10)	0 (0/10)	0 (0/10)
T + Cryo	70 (7/10)	29 (2/7)	100 (7/7)	29 (2/7)	30 (3/10)	20 (2/10)	20 (2/10)
C	20 (2/10)	50 (5/10)	100 (10/10)	50 (5/10)	40 (4/10)	30 (3/10)^*^	30 (3/10)^*^
C + PVS2	40 (4/10)	60 (6/10)	60 (6/10)	60 (6/10)	40 (4/10)	20 (2/10)	20 (2/10)
C + Cryo	90 (9/10)	100 (6/6)	100 (6/6)	100 (10/10)	70 (7/10)	70 (7/10)	70 (7/10)
C + (C + T)	50 (5/10)	60 (6/10)	100 (10/10)	60 (6/10)	60 (6/10)	50 (5/10)	40 (4/10)
C + (C + T) + PVS2	80 (8/10)	60 (6/10)	100 (10/10)	60 (6/10)	40 (4/10)	50 (5/10)	40 (4/10)
[C + (C + T)] + Cryo	100 (8/8)	70 (7/10)	100 (10/10)	70 (7/10)	100 (10/10)	70 (7/10)	70 (7/10)
Plants grown *in vitro* for three months that tested negative	45	40	75	44	43	33	31
Plants tested negative at first test, grown in the greenhouse for three months, and retested negative	45	40	75	44	43	31	29

* Plants grown in vitro were PVM-free, but one of these plants within the corresponding treatments tested positive for PVM later in plants grown in the greenhouse.

ST, shoot tip; Cryo, cryotherapy (liquid nitrogen treatment); PVS2, plant vitrification solution 2 (without liquid nitrogen exposure); +LN, liquid nitrogen treatment; T, thermotherapy; C, chemotherapy; PVM, Potato virus M; PVA, Potato virus A; and PVS, Potato virus S. Different letters in the same column indicate significant differences at *p* < 0.05 according to pairwise contrasts. Virus status of potato cultivars that underwent the in vitro therapies were assayed in in vitro plants have been grown for 3 months and confirmed again after grown the plants in greenhouse for 3 months. Numbers in parentheses are the number of plantlets showing a negative reaction for the virus/total samples analyzed by reverse-transcription PCR.

The effectiveness of virus eradication differed among the virus species: PVA was more readily eradicated than PVS and PVM, regardless of the method used (Table 3). Cryotherapy resulted in regrowth ranging from 42.2 to 55.4% across the three potato cultivars, however failed to eradicate PVS and PVM (Tables 2, 3). PVS2 treatment alone was not sufficient to eradicate any of the viruses. Shoot tips from “Tahi” recovered from cryotherapy were 20% PVA-free but still infected with PVS (Table 3).

Thermotherapy alone did not produce any virus-free plants in “Dunluce” and “Tahi”; meanwhile, in cultivar “V500,” one in 10 plants was free of PVS and PVM. Interestingly, two out of 10 thermotherapy-derived “V500” plants were free of PVM after 3 months in tissue culture but one subsequently tested positive for PVM after further plant growth in the greenhouse (Table 3).

Chemo-treated shoot tips produced similar rates of survival (about 100%) across the three potato cultivars with shoot regrowth varying from 48.3 to 90%, the lowest of which was for “V500” and the highest for “Dunluce” (Table 2). Chemotherapy alone was not always able to eradicate viruses; it was more efficient at eliminating PVA than PVS or PVM. After chemotherapy, “Tahi” initially infected with PVS and PVA had around 50% of plants free of virus. This compared to only 20% of plants free of virus for both “Dunluce” (which was only infected with PVS) and “V500” (which had a mixed infection of PVS and PVM). As noted for thermotherapy, three out of 10 “V500” plants submitted to chemotherapy were PVM-free after 3 months of *in vitro* culture post-regeneration. However, one of the progeny from this sample tested positive for PVM later in the greenhouse (Table 3).

Improvements on virus eradication were achieved through consecutive chemotherapy followed by combined chemotherapy and thermotherapy [C + (C + T)] treatments, with 50% of the evaluated “Dunluce” plants being free of PVS, 60% of the “Tahi” plants being free of PVS and PVA in mixed infection, and 40% of the “V500” plants being free of PVS and PVM in mixed infection (Table 3).

Thermotherapy or chemotherapy treatments followed by cryotherapy and the combination of all treatments affected shoot tip survival and regrowth in all three potato cultivars assessed (Table 2). When thermotherapy was combined with cryotherapy, the regrowth varied from 11.1% (“Tahi”) to 23.7% (“V500”). Chemotherapy in combination with cryotherapy resulted in a particularly low regrowth of 8.6% in “Tahi.” In general, shoot tips from treatments combined with either PVS2 (PVS2-treated shoot tips without LN exposure) or/and cryotherapy (PVS2 + LN exposure) treatments had the lowest regrowth (Table 2). Although regrowth was lower in shoot tips recovered after combining chemotherapy with cryotherapy, this method resulted in the highest frequencies of virus eradication in both single and mixed infections. All the “Tahi” plants recovered after chemotherapy combined with cryotherapy were free from both PVS and PVA, while nine out of 10 “Dunluce” plants evaluated were free from PVS and seven out of 10 “V500” plants were free from both PVS and PVM (Table 3).

The efficiency of virus eradication using thermotherapy followed by cryotherapy differed between virus species and cultivar. While 70% of the “Dunluce” plants evaluated were PVS-free, only 29% of the “Tahi” plants evaluated were free from both PVS and PVA, and 20% of the “V500” plants were free of PVS and PVM (Table 3).

Shoots exposed to chemotherapy followed by combined chemotherapy plus thermotherapy; then, cryotherapy {[C + (C + T)] + Cryo} had significantly decreased regrowth compared to the control or to shoots received individual treatments (Table 2). Although the shoot tip survival was relatively high (58.7–63.8%), most of the shoot tips produced just leaves with no evidence of a continued growth or turned into callus and failed to regenerate. The combination of all treatments resulted in shoot regrowth of 10.7–29% across the three potato cultivars with virus eradication rates of 70–100% (Table 3).

Virus-free plants, at low frequencies, could be found in shoots subjected to thermo- or/and chemo-therapy followed by PVS2 exposure for 60 min without freezing in LN (PVS2 treatment). However, the efficiency of virus eradication was much higher when shoot tips were thermo- or/and chemo-treated followed by LN exposure (Cryo; Table 3).

### Effect of PVS2 Exposure Duration on Shoot Tip Viability and Virus Eradication

Survival and regrowth of shoot tips decreased as durations of PVS2 treatment increased, for all three cultivars. Extended PVS2 exposure duration of 135 min resulted in the least shoot tip survival and regrowth, even without LN exposure (Tables 4, 5; Supplementary Tables 3–5).

**Table 4:** Effect of seven plant vitrification solution 2 (PVS2) exposure durations without (–LN) and with freezing in liquid nitrogen (+LN) on the survival and regrowth of shoot tips excised from *in vitro*-grown potato “Dunluce” infected with *Potato virus S*.

**LN**	**PVS2 time (min)**	**Survival (%)**	**Regrowth (%)**	***N***
–LN	5	100 a	96.8 a	63
	15	100 a	91.8 a	61
	60	98.4 ab	73.8 b	61
	90	96.7 abc	73.3 b	60
	105	91.8 bc	70.5 bc	61
	120	91.8 bc	55.7 cd	61
	135	47.5 e	9.8 f	61
+LN	5	78.8 d	22.7 e	66
	15	100 a	78.5 b	65
	60	94.6 bc	55.4 cd	74
	90	98.6 ab	56.5 cd	69
	105	89.1 cd	45.3 d	64
	120	90.5 cd	41.9 d	74
	135	41.7 e	3.3 f	60

–LN, cryotherapy procedure followed without liquid nitrogen exposure; +LN, liquid nitrogen treatment; PVS2, plant vitrification solution 2; and *N*, number of shoot tips used for each treatment. Different letters in the same column indicate significant differences at *p* < 0.05 according to pairwise contrasts.

**Table 5:** Effect of three plant vitrification solution 2 (PVS2) exposure durations without (–LN) and with freezing in liquid nitrogen (+LN) on survival and regrowth of shoot tips excised from *in vitro*-grown potato cultivars “Tahi” (infected with *Potato virus S* and *Potato virus A*) and “V500” (infected with *Potato virus S* and *Potato virus M*).

**LN**	“**Tahi**”				“**V500**”			
	**PVS2 time (min)**	**Survival (%)**	**Regrowth (%)**	***N***	**PVS2 time (min)**	**Survival (%)**	**Regrowth (%)**	***N***
–LN	5	100 a	100 a	60	5	100 a	96.7 a	61
	60	100 a	58.7 b	63	60	96.9 ab	58.5 b	65
	135	42.2 c	10.9 d	64	135	56.7 c	23.3 c	60
+LN	5	87.7 b	29.2 c	65	5	88.5 b	16.4 c	61
	60	77.1 b	44.3 bc	70	60	93.8 b	42.2 b	64
	135	41.3 c	3.2 d	63	135	46.0 c	4.8 d	63

–LN, cryotherapy procedure followed without liquid nitrogen exposure; +LN, liquid nitrogen treatment; PVS2, plant vitrification solution 2; and *N*, number of shoot tips used for each treatment. Different letters in the same column indicate significant differences at *p* < 0.05 according to pairwise contrasts.

Shoot tip survival of “Dunluce” was between 48 to 100% and 42 to 100% across the seven PVS2 exposure times for non-LN (without freezing in LN) treated and LN-treated (Cryo) explants, respectively (Table 4). Regrowth percentages in non-LN-treated shoot tips decreased from 97 to 3.3% as the PVS2 exposure time increased. In LN-treated shoot tips (Cryo), the maximum regrowth was 79% after 15 min of PVS2 exposure. Shoot tips exposed to PVS2 for a duration shorter or longer than 15 min showed reduced viability (Table 4). Although the regrowth of “Dunluce” shoot tips was high when incubated at the optimized PVS2 treatment duration (15 min) and exposed to LN, all recovered plants were infected with virus. Likewise, all shoot tips exposed to PVS2 without freezing in LN, were virus-infected regardless of the PVS2 exposure time. While the optimized vitrification solution exposure duration followed by LN treatment failed to eradicate PVS, one out of two plants tested free of this virus when a longer PVS2 exposure (135 min) was followed by LN treatment (Table 6). Longer PVS2 exposure followed by LN treatment (Cryo) resulted in regrowth of only two of out 60 treated shoot tips. The PVS was not able to be eradicated when shoot tips of cultivar “Dunluce” were exposed to PVS2 for a duration shorter than 135 min prior to LN treatment (Table 5).

**Table 6:** Effect of plant vitrification solution 2 (PVS2) exposure duration of 5, 60, and 135 min without (–LN) and with freezing in liquid nitrogen (+LN) on shoot regrowth level and percentage of potato plants from cultivars “Dunluce,” “Tahi,” and “V500” that were virus-free in a single or mix-infection.

**LN**	**PVS2 time (min)**	“**Dunluce**”		“**Tahi**”				“**V500**”			
		**Shoot regrowth (%)**	**PVS- free plants (%)**	**Shoot regrowth (%)**	**Virus-free plants (%)**			**Shoot regrowth (%)**	**Virus-free plants (%)**		
					**PVS**	**PVA**	**PVS- and PVA-free**		**PVS**	**PVM**	**PVS- and PVM-free**
–LN	5	96.8 a	0 (0/10)	100 a	0 (0/10)	0 (0/10)	0 (0/10)	96.7 a	0 (0/10)	0 (0/10)	0 (0/10)
	60	73.8 b	0 (0/10)	58.7 b	0 (0/10)	0 (0/10)	0 (0/10)	58.5 b	0 (0/10)	0 (0/10)	0 (0/10)
	135	9.8 f	0 (0/6)	10.9 d	0 (0/10)	0 (0/10)	0 (0/10)	23.3 c	0 (0/10)	0 (0/10)	0 (0/10)
+LN	5	22.7 e	0 (0/10)	29.2 c	0 (0/10)	0 (0/10)	0 (0/10)	16.4 c	0 (0/10)	0 (0/10)	0 (0/10)
	60	55.4 cd	0 (0/10)	44.3 bc	0 (0/10)	20 (2/10)	0 (0/10)	42.2 b	0 (0/10)	0 (0/10)	0 (0/10)
	135	3.3 f	50 (1/2)	3.2 d	50 (1/2)	100 (2/2)	50 (1/2)	4.8 d	33 (1/3)	33 (1/3)	33 (1/3)

–LN, cryotherapy procedure followed without liquid nitrogen exposure; +LN, liquid nitrogen treatment; PVS2, plant vitrification solution 2; PVM, Potato virus M; PVA, Potato virus A; and PVS, Potato virus S. Numbers in parentheses are the number of plantlets showing a negative reaction for the virus/total samples analyzed by reverse-transcription PCR. Different letters in the same column of shoot regrowth indicate significant differences at *P* < 0.05 according to pairwise contrasts.

There were no significant differences in regrowth between shoot tips of “V500” and “Tahi” that were immersed in LN and those without LN immersion (PVS2 treatment) for the PVS2 exposure durations of 60 and 135 min (Table 5). Shoot regrowth was higher in explants that were exposed to PVS2 for 5 min without freezing in LN (97–100%) than those that were immersed in LN (16–29%; Supplementary Tables 4, 5). All the shoot tips that were exposed to PVS2 without LN treatment remained virus-infected. As observed in “Dunluce,” all the shoot tips of “Tahi” and “V500” that were exposed to PVS2 without LN treatment remained virus-infected. Only the shoot tips that were exposed to PVS2 for 135 min and immersed in LN produced virus-free plants (Table 6). Although one plant from each cultivar was free of mixed virus infection, only two and three out of 63 treated shoot tips of “Tahi” and “V500” were recovered after cryotherapy, respectively. Shoot tips exposed to PVS2 for a duration shorter than 135 min followed by LN treatment failed to eradicate mixed infections of PVS plus PVA and PVS plus PVM in “V500” and “Tahi” cultivars, respectively. For “Tahi,” although both plants recovered from shoot tips exposed to 135 min in PVS2 and immersed in LN were free of PVA, only one of those was also free of PVS (Table 6).

All regenerated potato plantlets were successfully rooted *in vitro* and survived the acclimatization process. No visible morphological difference was apparent in the plants following 3 months’ growth in the greenhouse. Except for two “V500” plants, all the plants that were virus-free at the *in vitro* stage were found to have a virus-free status after 3 months in the greenhouse. The two “V500” plants (from mixed-infected with PVS and PVM) tested positive for PVM; one was derived from thermotherapy alone and the other from chemotherapy alone (Table 3).

### Comparative Assessment of Microtuber Production in the Virus-Free and Virus-Infected Potato Plants

**Figure 4:**
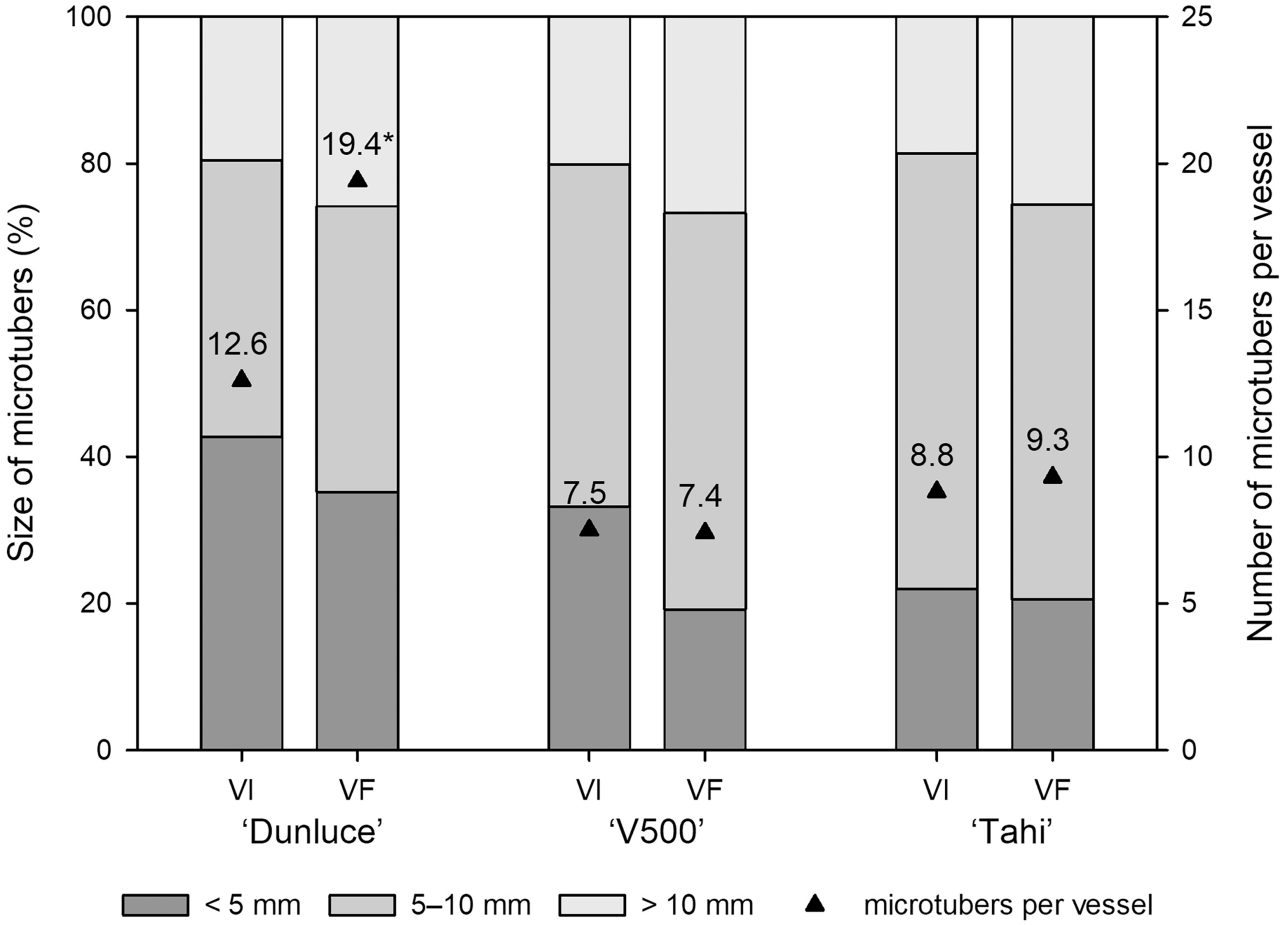
Effect of virus infection on proportion of microtuber size produced in potato cultivars “Dunluce” (infected with *Potato virus S*), “V500” (infected with *Potato virus S* and *Potato virus M*), and “Tahi” (infected with *Potato virus S* and *Potato virus A*) after 2 weeks in microtuber production media. *VI virus-infected plants; VF virus-free plants.* ^*^ indicates significant differences at p < 0.05 in microtuber numbers according to analysis of variance.

Each of the three potato cultivars consistently produced 7–19 microtubers of varied size per vessel (Figure 4). Single infection of PVS in “Dunluce” had a negative impact on microtuber production *in vitro*: the number of microtubers per vessel was significantly lower in PVS-infected plantlets (12.6) than in PVS-free plantlets (19.4; Figures 3, 4 and Supplementary Table 6). Although there were no significant differences in the number of microtubers per vessel and proportions of microtubers in each size class between virus-free and virus-infected “V500” and “Tahi,” there was a tendency for higher proportions of large microtubers (>10 mm) to be produced in virus-free plantlets (Supplementary Table 7). A minimal increment of 5% in larger sized microtubers (>10 mm) was achieved when virus-free shoots were used as the source of material for microtuber production. Cultivar “V500” produced almost twice the percentage of small-sized microtubers from virus-infected (38.3%) material compared with virus-free (20.3%) material (Figure 4).

## Discussion

Potato is highly susceptible to virus infections and viruses have long affected global potato production (Kreuze et al., 2020). Thus, considerable effort has been made to establish robust systems for obtaining and maintaining virus-free potato plants. The cultivation of virus-resistant plants might be a desirable and sustainable strategy to overcome this problem (Farnham and Baulcombe, 2006; Valkonen, 2008; Orbegozo et al., 2016). However, virus resistance is not easily achievable for most crops and viruses, and mutations in the viral genome or even mixed infection can overcome this resistance (García-Cano et al., 2006; Tian and Valkonen, 2013; Rubio et al., 2020). Therefore, the development of technologies to produce virus-free propagation material followed by the ongoing planting of high-healthy plants is still the most effective strategy for managing viruses in commercial production systems (Gong et al., 2019; Magyar-Tábori et al., 2021).

In this study, we combined several virus eradication methods (thermotherapy, chemotherapy, and cryotherapy) to efficiently eradicate single and mixed PVS, PVA, and PVM infection from virus-infected *in vitro* cultures of potato cultivars “Dunluce,” “Tahi,” and “V500”. We found that the combination of chemotherapy and cryotherapy (C + Cryo), or chemotherapy treatment prior to combining chemotherapy treatment with thermotherapy followed by cryotherapy {[C + (C + T)] + Cryo} resulted in high efficiency of PVS, PVA, and PVM eradication; 70–100% across the three potato cultivars tested. Though the regrowth percentage was low in shoots exposed to chemotherapy followed by cryotherapy (8.6–28.8%), high levels of virus-free plants were obtained. Indeed, plants of “Tahi” were 100% free from PVS and PVA, “Dunluce” were 90% PVS-free, and “V500” were 70% free from mixed infection of PVS and PVM. The combination of all treatments resulted in similar shoot regrowth (10.7–29%) across the three cultivars with virus eradication rates of between 70 and 100%: with rates for “Tahi” (mixed-infected with PVS and PVA) being lower than “V500” (mixed-infected with PVS and PVM) which was lower than “Dunluce” (PVS-infected). Although the efficacy of these two treatments in eradicating viruses was similar, incorporating thermotherapy was more time-consuming and required a temperature-controlled growth chamber; therefore, our study shows that the combination of chemotherapy and cryotherapy is a more appropriate strategy for eradication of PVS, PVM, and PVA from infected *in vitro* potato plants.

In this study, isolating and regrowing plants from 1 mm shoot tips (without any virus eradication treatment) completely failed to eradicate single infections of PVS, mixed infections of PVS and PVA, and PVS and PVM from infected *in vitro* potato shoots. In shoot tip culture, the size of excised shoot tip affects to the success of virus eradication (Bettoni et al., 2021a); our results confirm that the 1 mm shoot tips were not a suitable explant size for virus eradication. In addition, the virus location in the infected shoot tips may be a bottleneck to achieving efficient virus eradication by shoot tip culture (Wang et al., 2021). Previously, PLRV, PVY, PVS, and *Potato virus X* (PVX) have been eradicated by 0.2 mm shoot tip culture from *in vitro* diseased shoots (Wang et al., 2006; AlMaarri et al., 2012; Jianming et al., 2012; Zhang et al., 2019). Although 0.2 mm shoot tip culture could eradicate virus species, a proportion of recovered plants were still virus-infected, with eradication efficiency being related to the specific interaction between virus species and potato genotype. Improved virus eradication using shoot tip culture has been achieved when it is combined with thermotherapy (Waswa et al., 2017). High temperature treatments can prevent virus movement toward meristematic cells by inhibiting viral replication and degrading RNA and therefore, decreasing the viral particle load in infected shoot tips (Wang et al., 2008; Wang et al., 2018a). Wang et al. (2006) found that PLRV and PVY eradication frequencies increased from 56 to 90% and 62 to 93%, respectively, when 0.2 mm shoot tips were excised from shoots heat-treated at 36°C for 4 weeks, compared to shoot tips not subjected to heat treatment (thermotherapy). We found that thermotherapy involving alternating temperature of 28°C (8 h in dark) and 40°C (16 h in light) for 2 weeks followed by the excision of 1 mm shoot tips resulted in very low efficiency of virus eradication in “V500,” which was mixed-infected with PVS and PVM, and failed to eradicate either the single infection of PVS from “Dunluce” or the mixed infection of PVS and PVA from “Tahi.” Although 70% of “Tahi” shoots were free of PVA following this thermotherapy regime, none of the recovered plants were free of PVS. In general, prolonged heat treatment associated with high temperatures can increase the frequency of virus eradication, but at the same time reduces the viability of the treated explants, as host plants are often sensitive to these conditions (Zhao et al., 2018; Wang et al., 2018a; Farhadi-Tooli et al., 2022).

The combination of chemotherapy and cryotherapy reported here involved treatment of the shoots in medium containing 100 mg L^−1^ ribavirin for 4 weeks, followed by cryotherapy. Previously, Kushnarenko et al. (2017) have combined chemotherapy and cryotherapy procedures to eradicate virus species from *in vitro* shoots of potato. In their study, PVM and PVS were successfully eradicated from potato cultivars “Tamyr” and “Nartau” after three subcultures (45 days each; total of 135 days) on medium with 100 mg L^−1^ ribavirin followed by cryotherapy. In our study, in addition to PVM and PVS, we also successfully eradicated PVA by combining chemotherapy and cryotherapy. Since our procedure did not require such a long period of culture on ribavirin, it could provide a higher throughput and more cost effective technique, as well as reducing the potential for somaclonal variation through reducing the number of culture steps. In our study, 1-mm apical shoot tips were excised from plants treated with ribavirin for 4 weeks, then subjected to droplet-vitrification cryotherapy using 60 min PVS2 exposure at 22°C; in contrast, Kushnarenko et al. (2017) used slightly larger shoot tips (1.5–2.0 mm), a different cryotherapy technique (vitrification) and shorter PVS2 exposure (30 min). These differences might explain why a longer ribavirin treatment was necessary for the eradication of PVM and PVS in the study of Kushnarenko et al. (2017). We note that chemotherapy treatment alone could eliminate viruses from all three cultivars but with low efficacy; this could be significantly improved by combining chemotherapy with cryotherapy, specifically from 20 to 90% in “Dunluce,” 50 to 100% in “Tahi,” and 20 to 70% in “V500.”

In recent years, shoot tip cryotherapy has proven to be an effective method to eradicate viruses from *in vitro* infected plants in multiple species of economic importance (Cui et al., 2015; Pathirana et al., 2015; Bettoni et al., 2018, 2019a, 2021a; Bi et al., 2018; Souza et al., 2020; Farhadi-Tooli et al., 2022; Wang et al., 2022a). Cryotherapy makes use of LN exposure (−196°C) to selectively destroy vacuolated and differentiated cells that are known to harbor viruses within shoot tips (Wang et al., 2009; Wang and Valkonen, 2009). In this study, cryotherapy (60 min PVS2 exposure + LN) alone failed to eradicate virus in “Dunluce” singly infected with PVS or mixed infections of PVS and PVA in “Tahi,” and PVS and PVM in “V500.” Similarly, Kushnarenko et al. (2017) found that cryotherapy alone did not produce any potato plants free from mixed infection of PVS and PVM. Working on six PVS-infected potato cultivars, Zhang et al. (2019) found that successful PVS eradication using cryotherapy was cultivar dependent. About 17% of the “Iverpotet/Smaragd” cryo-recovered plants were PVS-free, while all of the other potato cultivars were still PVS infected after cryotherapy. Li et al. (2018a) also reported that cryotherapy alone completely failed to generate PVS and *Potato spindle tuber viroid* (PSTVd)-free plants from *in vitro* infected potato “Zihuabai” shoots. In our study, although cryotherapy resulted in low efficiency (20%) of PVA eradication in “Tahi,” the cryo-recovered plants were still PVS-infected. Recent research has shown that not all viruses can be eradicated using shoot tip cryotherapy, particularly in those that are able to infect regions within the meristem (Wang et al., 2008, 2018b, 2022a; Li et al., 2016, 2018a; Zhao et al., 2018; Mathew et al., 2021). The ongoing presence of PVS and PVM after cryotherapy might be because these viruses can be located very close to the meristem; hence, it is possible for a small portion of the infected cells to remain alive following cryo-treatment, meaning the regenerated plants will remain virus-infected.

Even though cryotherapy techniques are based on cryopreservation, unlike cryopreservation, cryotherapy protocols should seek to eliminate the maximum portion of differentiated and infected cells, particularly in plant species infected with viruses that are difficult to eradicate such as PVS and PVM. This is a balancing act: a strategy to improve virus eradication could be to use either shorter or longer PVS2 exposure durations than the optimal, but this may result in fewer cells from which to recover plants after LN exposure (Bi et al., 2018; Bettoni et al., 2019a, 2021b; Souza et al., 2020; Wang et al., 2022b). Working on *Vitis vinifera* Cabernet Sauvignon, Bi et al. (2018) found that *Grapevine leafroll-associated virus-3* (GLRaV-3) was eradicated from diseased shoot tips independent of PVS2 exposure duration (50–100 min). Although, there was no influence of PVS2 exposure duration on GLRaV-3 eradication, it was evident that the long exposure to PVS2 (100 min) significantly reduced the percentage of cells surviving in the apical dome and leaf primordia. GLRaV-3 is a phloem-limited virus and, therefore, it might explain why Bi et al. (2018) did not observe any difference in virus eradication across the range of PVS2 times, even though longer PVS2 exposure duration of 100 min resulted in fewer cells that recover after LN exposure. In our study, the viability of shoot tips and the ability of cryotherapy to produce virus-free plants was directly associated with duration of exposure to PVS2. Extended PVS2 exposure durations of 135 min at room temperature followed by LN treatment resulted in very low shoot tip regrowth of 3.2–4.8% across the three potato cultivars; however, one out of 60 cryo-treated shoot tips from each cultivar did regenerate a virus-free plant irrespective of whether there was a single or mixed infection of PVS, PVM, and PVA. In vitrification-based methods, tolerance to freezing in LN is achieved through an osmotic process when shoot tips are placed in highly concentrated vitrification solutions (Volk et al., 2006). In addition to any LN effect, the long PVS2 exposure duration of 135 min used in this study might have selectively weakened and killed cells that harbored viruses through osmotic and chemical stress and therefore, influenced the production of virus-free plants after cryotherapy. It is worth noting that although cryotherapy alone (135 min PVS2 + LN) was able to produce at least one virus-free plant in each potato cultivar, when combined with chemotherapy it resulted in higher shoot regrowth and virus eradication frequencies.

Results obtained in the present study clearly demonstrate that PVS, PVA, and PVM in single or mixed infection negatively affect microtuber production of *in vitro* cultures of “Dunluce,” “Tahi,” and “V500.” This finding is consistent with results reported by Li et al. (2013, 2018b) and Zhang et al. (2019). Li et al. (2013) and Zhang et al. (2019) also noted that the co-infections of PLRV + PVY and PLRV + PVS and the triple infection of PVY and PVX + PVS could lead to negative effects on vegetative growth of potato *in vitro*. Negative effects on tuber yield of single or mixed infections of PVX + PVS have also been reported in greenhouse- and field-grown potato plants (Wright, 1977; Nyalugwe et al., 2012; Gong et al., 2019). Negative effects of virus infection on tuber yield of field-grown potato plants have been well documented and have long been a constraint for sustainable potato production (Brunt, 2011; Wang et al., 2011). This highlights the need to produce healthy and virus-free seed potatoes to support the potato industry.

To guard against possible false negatives, the virus status of plants that underwent *in vitro* therapies should be assessed for a second time after the plants have grown in the greenhouse: one should not rely just on the test result for *in vitro* plants. Virus titer may have been reduced and virus particles may occur in tissues that are not sampled for virus testing at the *in vitro* stage in some treatments, resulting in false negative, as we have shown in two “V500” plants (PVM-infected) derived from thermotherapy and chemotherapy treatments in this research and previously in raspberry and apple plants infected with *Raspberry bushy dwarf virus* and *Apple hammerhead viroid*, subjected to chemotherapy treatment (Mathew et al., 2021), and combined thermotherapy with cryotherapy (Bettoni et al., 2022), respectively. Therefore, repeat virus testing on progenies growing in the greenhouse and later in the field is recommended as standard best practice to confirm virus-free status of the plant materials.

In addition to presenting a protocol for virus eradication, we also identified an effective droplet-vitrification cryopreservation method to preserve potato shoot tips for long-term storage, using *in vitro* plants as source materials. Nodal sections from this material were placed on BM medium for 1 week to generate shoots from which uniform 1 mm apical shoots could be excised. Shoot tips were precultured on media enriched with sucrose, followed by PVS2 treatment for 15 min at 22°C prior to LN exposure. Shoot tips were warmed in unloading solution and placed on intermediate recovery medium overnight and, finally, transferred to recovery medium. This method resulted in high regrowth (79%) in potato cultivar “Dunluce.” Availability of a simple and efficient cryopreservation protocol that results in high levels of viability (≥ 40% after LN exposure), such as the one presented here, may facilitate the technology transfer between laboratories and the implementation of cryopreserved base collections (Volk et al., 2016; Bettoni et al., 2019b, 2021b).

Our study identified procedures that resulted in high frequencies of eradication of PVS, PVA, and PVM viruses from infected potato cultivars. The consistency of the results across three cultivars with single or mixed infections suggests that these procedures have great potential to assist the production and supply of virus-free planting materials for the potato industry. Furthermore, it might also be a valuable tool to support the global exchange of germplasm underpinning breeding activities.

## Data Availability Statement

The datasets presented in the study are either included in the article or in the Supplementary Material: further inquiries can be directed to the corresponding author.

## Author Contributions

JB, LM, RP, CW, DH, and JN conceived and designed the research and internal/external liaison with partners and service providers for contracts and IP rights protection. JB and LM planned and performed the experiments. JB, LM, and CW collected plant material for diagnosis. JB collected and interpreted the data, conducted the microtuber experiment, acclimatized and managed the plants in the greenhouse, prepared tables and figures, and wrote the original manuscript. DH wrote the diagnosis protocol. AM conducted the statistical analysis and helped prepare the figures. SK and JT developed the primers for virus diagnosis. All authors contributed to the article and approved the submitted version.

## Funding

This research was funded by the New Zealand Institute for Plant and Food Research Limited SSIF funding (1972—“Breeding Technology Development”).

## Conflict of Interest

JB LM, RP, CW, DH, AM, and JN were employed by The New Zealand Institute for Plant and Food Research Limited.

The remaining authors declare that the research was conducted in the absence of any commercial or financial relationships that could be construed as a potential conflict of interest.

## Publisher’s Note

All claims expressed in this article are solely those of the authors and do not necessarily represent those of their affiliated organizations, or those of the publisher, the editors and the reviewers. Any product that may be evaluated in this article, or claim that may be made by its manufacturer, is not guaranteed or endorsed by the publisher.

## Footnotes

^1^ https://sg.idtdna.com

^2^ https://www.slipstream-automation.co.nz

^3^ ThermoFisher.com

^4^ https://lifescience.roche.com/
