# Supplementary material for: Eradication of Potato Virus S, Potato Virus A, and Potato Virus M From Infected in vitro-Grown Potato Shoots Using in vitro Therapies
Source: Front Plant Sci. 2022 May 19;13:878733. doi: 10.3389/fpls.2022.878733 (PMC9161163; doi:10.3389/fpls.2022.878733)
Supplement: Supplementary file 1 [file Data_Sheet_1.docx]

Supplementary Material

# Supplementary Tables

**TABLE 1.** Predicted mean percentage and 95% confidence intervals for survival of potato cultivars ‘Dunluce’, ‘Tahi’ and ‘V500’ in response to 12 treatments.

| **Treatment** | **Survival (%)** | | | | | | | | | | | | | |
| --- | --- | --- | --- | --- | --- | --- | --- | --- | --- | --- | --- | --- | --- | --- |
|  | **N** | ‘**Dunluce**’ | | |  | **N** | ‘**Tahi**’ | | |  | **N** | ‘**V500**’ | | |
|  |  | **Mean** | | **95% C.I.** |  |  | **Mean** | | **95% C.I.** |  |  | **Mean** | | **95% C.I.** |
| ST (control) | 62 | 100 | (94.2, 100) | |  | 61 | 100 | (94.1, 100) | |  | 60 | 100 | (94.0, 100) | |
| PVS2 | 61 | 98.4 | (88.2, 99.8) | |  | 63 | 100 | (94.3, 100) | |  | 65 | 96.9 | (87.7, 99.3) | |
| Cryo | 74 | 94.6 | (85.8, 98.1) | |  | 70 | 77.1 | (65.2, 85.9) | |  | 64 | 93.8 | (83.8, 97.8) | |
| T | 62 | 100 | (94.2, 100) | |  | 60 | 98.3 | (88.0, 99.8) | |  | 61 | 100 | (94.1, 100) | |
| T + PVS2 | 72 | 95.8 | (87.2, 98.7) | |  | 72 | 94.4 | (85.5, 98.0) | |  | 63 | 87.3 | (75.9, 93.8) | |
| T + Cryo | 78 | 67.9 | (56.2, 77.8) | |  | 63 | 76.2 | (63.5, 85.5) | |  | 76 | 80.3 | (69.2, 88.1) | |
| C | 60 | 98.3 | (88.0, 99.8) | |  | 60 | 100 | (94.0, 100) | |  | 60 | 100 | (94.0, 100) | |
| C + PVS2 | 67 | 88.1 | (77.2, 94.1) | |  | 67 | 92.5 | (82.6, 97.0) | |  | 70 | 95.7 | (86.9, 98.7) | |
| C + Cryo | 80 | 55.0 | (43.5, 66.0) | |  | 70 | 58.6 | (46.1, 70.0) | |  | 76 | 80.3 | (69.2, 88.1) | |
| C + (C +T) | 61 | 98.4 | (88.2, 99.8) | |  | 60 | 100 | (94.0, 100) | |  | 60 | 100 | (94.0, 100) | |
| [C + (C +T)] + PVS2 | 66 | 78.8 | (66.6, 87.4) | |  | 64 | 93.8 | (83.8, 97.8) | |  | 60 | 100 | (94.0, 100) | |
| [C + (C +T)] + Cryo | 75 | 58.7 | (46.7, 69.7) | |  | 74 | 56.8 | (44.7, 68.1) | |  | 69 | 63.8 | (51.2, 74.7) | |

*N number of shoot tips used for each treatment, ST shoot tip, Cryo cryotherapy (liquid nitrogen treatment), PVS2 plant vitrification solution 2 (without liquid nitrogen exposure), T thermotherapy, C chemotherapy, C.I. confidence intervals.*

**TABLE 2.** Predicted mean percentage and 95% confidence intervals for regrowth of potato cultivars ‘Dunluce’, ‘Tahi’ and ‘V500’ in response to 12 treatments.

| **Treatment** | **Regrowth (%)** | | | | | | | | | | | | | |
| --- | --- | --- | --- | --- | --- | --- | --- | --- | --- | --- | --- | --- | --- | --- |
|  | **N** | ‘**Dunluce**’ | | |  | **N** | ‘**Tahi**’ | | |  | **N** | ‘**V500**’ | | |
|  |  | **Mean** | | **95% C.I.** |  |  | **Mean** | | **95% C.I.** |  |  | **Mean** | | **95% C.I.** |
| ST (control) | 62 | 96.8 | (87.2, 99.2) | |  | 61 | 98.4 | (88.5, 99.8) | |  | 60 | 100 | (94.0, 100) | |
| PVS2 | 61 | 73.8 | (60.7, 83.7) | |  | 63 | 58.7 | (45.6, 70.7) | |  | 65 | 58.5 | (45.6, 70.3) | |
| Cryo | 74 | 55.4 | (43.4, 66.8) | |  | 70 | 44.3 | (32.6, 56.6) | |  | 64 | 42.2 | (30.2, 55.2) | |
| T | 62 | 93.5 | (83.3, 97.7) | |  | 60 | 80.0 | (67.3, 88.6) | |  | 61 | 50.8 | (37.9, 63.7) | |
| T + PVS2 | 72 | 47.2 | (35.5, 59.3) | |  | 72 | 43.1 | (31.6, 55.3) | |  | 63 | 33.3 | (22.4, 46.5) | |
| T + Cryo | 78 | 12.8 | (6.8, 22.8) | |  | 63 | 11.1 | (5.2, 22.2) | |  | 76 | 23.7 | (15.1, 35.1) | |
| C | 60 | 90.0 | (78.7, 95.6) | |  | 60 | 80.0 | (67.3, 88.6) | |  | 60 | 48.3 | (35.4, 61.5) | |
| C + PVS2 | 67 | 56.7 | (44.1, 68.6) | |  | 67 | 40.3 | (28.8, 53.0) | |  | 70 | 41.4 | (30.0, 53.9) | |
| C + Cryo | 80 | 28.8 | (19.5, 40.2) | |  | 70 | 8.6 | (3.7, 18.5) | |  | 76 | 19.7 | (11.9, 30.8) | |
| C + (C +T) | 61 | 88.5 | (77.1, 94.6) | |  | 60 | 81.7 | (69.1, 89.9) | |  | 60 | 41.7 | (29.4, 55.1) | |
| [C + (C +T)] + PVS2 | 66 | 48.5 | (36.1, 61.0) | |  | 64 | 65.6 | (52.6, 76.7) | |  | 60 | 45.0 | (32.4, 58.3) | |
| [C + (C +T)] + Cryo | 75 | 10.7 | (5.2, 20.5) | |  | 74 | 17.6 | (10.2, 28.6) | |  | 69 | 29.0 | (19.1, 41.4) | |

*N number of shoot tips used for each treatment, ST shoot tip, Cryo cryotherapy (liquid nitrogen treatment), PVS2 plant vitrification solution 2 (without liquid nitrogen exposure), T thermotherapy, C chemotherapy, C.I. confidence intervals.*

**TABLE 3.** Predicted mean percentage and 95% confidence intervals for survival and regrowth of ‘Dunluce’ potato tissue after treatment with PVS2 for different amounts of time.

|  | **PVS2** | **Survival** | |  | **Regrowth** | |  |
| --- | --- | --- | --- | --- | --- | --- | --- |
| **LN** | **Time (min)** | **Mean** | **95% C.I.** |  | **Mean** | **95% C.I.** | ***N*** |
| -LN | 5 | 100 | (94.3, 100) |  | 96.8 | (87.5, 99.3) | 63 |
|  | 15 | 100 | (94.1, 100) |  | 91.8 | (81.2, 96.7) | 61 |
|  | 60 | 98.4 | (88.4, 99.8) |  | 73.8 | (60.8, 83.6) | 61 |
|  | 90 | 96.7 | (86.9, 99.2) |  | 73.3 | (60.2, 83.3) | 60 |
|  | 105 | 91.8 | (81.2, 96.7) |  | 70.5 | (57.3, 80.9) | 61 |
|  | 120 | 91.8 | (81.2, 96.7) |  | 55.7 | (42.6, 68.1) | 61 |
|  | 135 | 47.5 | (34.9, 60.5) |  | 9.8 | (4.3, 20.8) | 61 |
| +LN | 5 | 78.8 | (66.7, 87.3) |  | 22.7 | (13.9, 34.9) | 66 |
|  | 15 | 100 | (94.4, 100) |  | 78.5 | (66.3, 87.1) | 65 |
|  | 60 | 94.6 | (85.9, 98.0) |  | 55.4 | (43.5, 66.7) | 74 |
|  | 90 | 98.6 | (89.6, 99.8) |  | 56.5 | (44.1, 68.1) | 69 |
|  | 105 | 89.1 | (78.2, 94.9) |  | 45.3 | (33.1, 58.1) | 64 |
|  | 120 | 90.5 | (80.9, 95.6) |  | 41.9 | (30.8, 53.9) | 74 |
|  | 135 | 41.7 | (29.5, 55.0) |  | 3.3 | (0.8, 13.1) | 60 |

*-LN cryotherapy procedure followed without liquid nitrogen exposure, +LN liquid nitrogen treatment, PVS2 plant vitrification solution 2, N number of shoot tips used for each treatment, C.I. confidence intervals.*

**TABLE 4.** Predicted mean percentage and 95% confidence intervals for survival and regrowth of ‘Tahi’ potato tissue after treatment with PVS2 for different amounts of time.

|  | **PVS2** | **Survival** | |  | **Regrowth** | |  |
| --- | --- | --- | --- | --- | --- | --- | --- |
| **LN** | **Time (min)** | **Mean** | **95% C.I.** |  | **Mean** | **95% C.I.** | ***N*** |
| -LN | 5 | 100 | (94.0, 100) |  | 100 | (94.0, 100) | 60 |
|  | 60 | 100 | (94.3, 100) |  | 58.7 | (44.9, 71.3) | 63 |
|  | 135 | 42.2 | (29.6, 55.9) |  | 10.9 | (4.9, 22.7) | 64 |
| +LN | 5 | 87.7 | (75.8, 94.2) |  | 29.2 | (18.6, 42.8) | 65 |
|  | 60 | 77.1 | (64.5, 86.3) |  | 44.3 | (32.0, 57.3) | 70 |
|  | 135 | 41.3 | (28.7, 55.1) |  | 3.2 | (0.7, 13.6) | 63 |

*-LN cryotherapy procedure followed without liquid nitrogen exposure, +LN liquid nitrogen treatment, PVS2 plant vitrification solution 2, N number of shoot tips used for each treatment, C.I. confidence intervals.*

**TABLE 5.** Predicted mean percentage and 95% confidence intervals for survival and regrowth of ‘V500’ potato tissue after treatment with PVS2 for different amounts of time.

|  | PVS2 | Survival | |  | Regrowth | |  |
| --- | --- | --- | --- | --- | --- | --- | --- |
| LN | Time | Mean | 95% C.I. |  | Mean | 95% C.I. | *N* |
| -LN | 5 | 100 | (94.1, 100) |  | 96.7 | (86.1, 99.3) | 61 |
|  | 60 | 96.9 | (86.8, 99.3) |  | 58.5 | (44.9, 70.9) | 65 |
|  | 135 | 56.7 | (42.6, 69.8) |  | 23.3 | (13.5, 37.2) | 60 |
| +LN | 5 | 88.5 | (76.3, 94.9) |  | 16.4 | (8.4, 29.4) | 61 |
|  | 60 | 93.8 | (83.0, 97.9) |  | 42.2 | (29.6, 55.9) | 64 |
|  | 135 | 46.0 | (33.0, 59.7) |  | 4.8 | (1.4, 15.4) | 63 |

*-LN cryotherapy procedure followed without liquid nitrogen exposure, +LN liquid nitrogen treatment, PVS2 plant vitrification solution 2, N number of shoot tips used for each treatment, C.I. confidence intervals.*

**TABLE 6.** Mean number of potato microtubers produced from virus-free and virus-infected cultivars.

| **Cultivar** | **Virus-free** | **Virus-infected** | **SEM** | **P-value** |
| --- | --- | --- | --- | --- |
| ‘Dunluce’ | 19.4 | 12.6 | (0.91) | 0.001 |
| ‘Tahi’ | 9.3 | 8.8 | (0.42) | 0.43 |
| ‘V500’ | 7.4 | 7.5 | (1.32) | 0.95 |

*SEM pooled standard error of the mean.*

*P-values are from one-way ANOVA.*

**TABLE 7.** Predicted mean percentage and 95% confidence intervals for proportion (%) of microtuber size from virus-free and virus-infected source material from a binomial generalized linear model.

|  | **Microtuber size** | **Virus-free** | |  | **Virus-infected** | |  |
| --- | --- | --- | --- | --- | --- | --- | --- |
| **Cultivar** |  | **Proportion**  **(numbers of microtubers*)** | **95% C.I.** |  | **Proportion**  **(numbers of microtubers*)** | **95% C.I.** | **P-value** |
| ‘Dunluce’ | < 5 mm | 35.5 (6.9) | (29.0, 42.5) |  | 41.6 (5.3) | (33.3, 50.4) | 0.25 |
|  | 5–10 mm | 38.7 (7.5) | (30.5, 47.6) |  | 37.6 (4.7) | (27.8, 48.6) | 0.87 |
|  | > 10 mm | 25.8 (5.0) | (21.0, 31.3) |  | 20.8 (2.6) | (15.5, 27.3) | 0.20 |
| ‘Tahi’ | < 5 mm | 21.6 (2.0) | (12.8, 34.1) |  | 24.3 (2.1) | (14.7, 37.4) | 0.72 |
|  | 5–10 mm | 54.1 (5.0) | (44.5, 63.3) |  | 57.1 (5.0) | (47.3, 66.4) | 0.63 |
|  | > 10 mm | 24.3 (2.3) | (18.4, 31.4) |  | 18.6 (1.6) | (13.2, 25.4) | 0.19 |
| ‘V500’ | < 5 mm | 20.3 (1.5) | (8.5, 41.4) |  | 38.3 (2.9) | (21.3, 58.9) | 0.16 |
|  | 5–10 mm | 52.5 (3.9) | (35.3, 69.2) |  | 40.0 (3.0) | (24.6, 57.6) | 0.29 |
|  | > 10 mm | 27.1 (2.0) | (18.9, 37.2) |  | 21.7 (1.6) | (14.4, 31.3) | 0.37 |

** Mean number of microtuber in each proportion of microtuber size.*

*C.I. confidence intervals.*

*P-values are from binomial* *generalised linear models.*
